# Concerns raised by people treated for head and neck cancer: a secondary analysis of audiotaped consultations in a health services follow-up clinic

**DOI:** 10.1007/s00520-023-08059-w

**Published:** 2023-10-04

**Authors:** Corrie Dicks, Simon N. Rogers, Anastasios Kanatas, Derek Lowe, Calum McHale, Gerry Humphris

**Affiliations:** 1 Division of Population and Behavioural Sciences, Medical School, University of St Andrews, St Andrews, KY16 9TF UK; 2 Evidence-Based Practice Research Centre (EPRC), Faculty of Health and Social Care, Edge Hill University, St Helens Road, Ormskirk, L39 4QP UK; 3 Maxillofacial Department, Wirral University Teaching Hospital, Arrowe Park, CH49 5PE UK; 4 Leeds Teaching Hospitals and St James Institute of Oncology, Leeds Dental Institute and Leeds General Infirmary, Leeds, UK; 5 Astraglobe Ltd., 24 Trinity Place, Mossley, Congleton, Cheshire, CW12 3JB UK

**Keywords:** Prompt sheets, Oncology, Communication, Psychological intervention, coding transcripts, multi-level modelling

## Abstract

**Purpose:**

People treated for head and neck cancer (HNC) face various barriers in communicating concerns with consultants. Our aim was to investigate the number of concerns raised between patients using the Patient Concerns Inventory (PCI) and those who did not. The PCI is a 57-item prompt list used in routine HNC follow-up clinics. Additionally, we aimed to examine whether who initiated the concerns differed between groups and the factors that may predict this initiation.

**Methods:**

Secondary data analysis included 67 participants across 15 HNC consultants from specialist cancer centres in Liverpool and Leeds. Seven consultants utilised the PCI and eight did not, assigned by preferential and random assignment.

**Results:**

Patients in the PCI group raised on average 2.5 more concerns than patients in the non-PCI group (*p* < .001). There was no significant relationship between group and who initiated the first concern (*p* = .28). A mixed-effects logistic regression was found to significantly predict who initiated the first concern in consultations (*p* < .05).

**Discussion:**

The number of concerns raised by patients increased when the PCI was introduced pre-HNC consultation. A number of factors were shown to predict the number of concerns raised in consultations by both patient and consultant. As concerns may not be raised further following the concern mentioned, we propose that the discussion of concerns needs to be maintained by the clinician throughout the consultation and not solely at the start.

**Conclusion:**

The PCI promoted the sharing of concerns in follow-up consultations between patient and consultant.

**Supplementary Information:**

The online version contains supplementary material available at 10.1007/s00520-023-08059-w.

## Introduction

Post-treatment head and neck cancer (HNC) consultations constitute an essential aspect of patient care, where improved communication has meaningful effects on the patient-doctor relationship and overall patient satisfaction [1–3]. Dimoska et al. suggests that a patient-centred approach within oncology consultations can be enhanced through the use of prompt lists [4]. Prompt lists used to encourage HNC patients to actively participate in discussions can enhance patient understanding and promote increased patient autonomy [4]. There are several positive psychological outcomes associated with increased patient participation including improved patient satisfaction with consultations, reduced levels of anxiety and distress, and increased overall symptom resolution [5, 6].

In oncology, general prompt tools, such as questions prompt lists (QPL), may lack sensitivity to issues specific to certain cancer types [7]. Patients with HNC are exposed to multiple physical and psychological challenges, such as impacted speech or fear of cancer recurrence [8]. Consequently, cancer patients value the opportunity to discuss their health concerns with health professionals during consultations [9]. Where patients voice less concerns, they experience worsening of symptoms, increased patient anxiety and healthcare visits, and reduced patient satisfaction [10].

Despite being an important aspect of consultations, patients face several barriers in expressing concerns [11–14]. Some health care providers do not openly invite patients to express their concerns [14, 15]. Additionally, patients perceive healthcare providers to have limited time in consultations and that expressing multiple concerns may impact on their relationship with their healthcare providers. [11].

The Patient Concern Inventory (PCI) [16] is a 57-item checklist developed to aid discussion about patient concerns in routine HNC follow-up clinics. The PCI covers physical, social, psychological, emotional, treatment related, and other concerns. Patients complete the PCI pre-consultation by selecting items of personal importance to them, which then guide the consultation discussion [8, 17]. Patients can also select specific healthcare professionals that they wish to meet, facilitating signposting to healthcare providers who can best offer support and advice for their concerns [18]. The PCI has been shown to have improve patient reported quality of life, as well as reduce the social-emotional impact of cancer and levels of patient distress [8, 19]. Furthermore, the PCI has been shown to be easily integrated into routine clinical practice without significantly prolonging consultation time [8, 19].

Despite the growing evidence that the PCI can facilitate discussion about patient concerns, as well as improved health-related outcomes, the precise impact that the PCI has on the consultation communication process is not fully understood. For example, it is not clear whether consultants raise the issue of concerns initially with the patient, or whether patients take the initiative to highlight their concerns without clinician prompting. A more detailed understanding of the effect of the PCI on consultation discussions may facilitate improvements in the way the PCI is administered or indicate more focused training that healthcare providers may receive on PCI application.

The aim of the present study is to investigate the influence of the PCI on communication processes regarding patient concerns during HNC follow-up consultations. Specific objectives include:

1. To investigate differences in the number of concerns raised in HNC follow-up consultations where patients completed the PCI prior to the consultation compared to consultations where patients did not complete the PCI.
2. To investigate whether the person who initiates the discussion surrounding concerns (consultant or patient) differs in the PCI and non-PCI groups and what factors may predict the initiation of discussion about patient concerns.

## Methods

### Study design

This paper reports on a secondary analysis of data collected during a pragmatic cluster-controlled trial conducted in two UK cancer centres [19]. Audio recordings of routine HNC follow-up consultations between HNC patients and consultants were analysed. Consultants had been randomised so that their patients did or did not complete the PCI prior to their consultation. Audio recordings were coded, focusing specifically on discussion about patient concerns as well as who (patient or consultant) raised patient concerns for discussion.

### Participants

A power analysis (*rsquared* routine in STATA [20]) estimated that 65 participants would be required to conduct a successful multiple regression model with 5 covariates at 80% power with alpha set to 0.05 assuming the total percent explanation of the dependent variable (number of concerns discussed in the consultation per patient) was 18%. The current study included secondary data of 67 HNC patients (46 male, 21 female) aged 28–84 (mean age: 60.01, sd: 10.6) that had previously been collected in a pragmatic cluster-controlled trial conducted at UK Cancer Centres [19]. The study also included 15 HNC consultants based in tertiary HNC units in the University Teaching Hospitals in Liverpool and Leeds. Patients who had been treated curatively for primary HNC were included regardless of disease site, cancer stage, or type of treatments undergone. Patient participants were excluded if they were treated palliatively or for recurrence, or had a history of cognitive impairment, psychosis, or dementia [8].

### Materials and apparatus

Audio tapes were routinely collected as part of the original trial procedure to check fidelity, in addition to enable detailed analysis of verbatim interaction. Secondary data were stored and backed up on encrypted USBs. Audio files were strictly accessed on a password protected PC within the secure office. Audio file coding was conducted using Microsoft Excel. During primary data collection, all patients were provided with the following pre-consultations questionnaires (results reported elsewhere): UW-QOLv4 [21], Distress Thermometer [22], EQ-5d-5L [23], and in the intervention group patients were provided with the PCI. HRQOL and PCI data were collected electronically using desktops, tablets, or iPADs in all cases except from one non-PCI consultant who gathered data using paper-based materials.

### Procedure

Secondary data analysed originally consisted of 68 anonymised audio recordings from 67 patients across 15 HNC consultants. Two audio files were removed from the data set due to incomprehensibility, as well as the second audio file of one patient who was recorded twice. Analysis therefore consisted of 65 audio files from 65 patients.

Consultants had previously been assigned to either the intervention (PCI) or control (non-PCI) group [19]. Consultants in the PCI group (*n* = 8) incorporated the PCI into their trial clinics. The remaining 7 consultants did not use the PCI nor any other form of prompt list. Consultants who had a preference of being in the PCI group or non-PCI group were assigned to either group according to their preference, with consultants holding no preference being randomised. This approach was applied to reduce the possibility of ‘PCI-sceptics’ dominating the intervention group and ‘PCI-enthusiasts’ the control group [19].

Patients were assigned to either PCI or non-PCI groups dependant on their consultant, where 36 patients used the PCI and 31 patients did not. In collection of the primary data eligible patients agreed to complete research questionnaires pre-consultation and agreed to their clinical data being used [19]. For the current study, only data gathered from the PCI were analysed.

The three audio tapes removed were of patients in the non-PCI group, resulting in there being 29 non-PCI patients. No participants were blind to their group allocation. After recording of HNC consultations, audio files were anonymised at baseline and patient identifying details were redacted by the Chief Investigator to ensure patient anonymity prior to being encountered by the current researchers.

The audio files were listened to and coded using a system specially devised for the current study (Table 1). Details of the coding scheme have been described (Appendix 1.1, 1.2). Coding was conducted using Excel, where a Patient File and Concerns File was created. In the Patient File each patient was assigned a row of data (*n* = 65). In the Concerns File every concern that was either pre-selected on the PCI or mentioned within PCI and non-PCI consultations was assigned a row of data (*n* = 329). Concerns that were pre-selected though not discussed in the consultation were removed before analysis was conducted, leaving 260 concerns within the Concerns File.

**Table 1:** Data coded in patient and concern files (*n* = 65)

Patient File	Concerns File
Trial Group	Trial Group
PCI	PCI
Non-PCI	Non-PCI
Audio Tape Label Name	Audio Tape Label Name
Patient Number	Patient Number
Patient Age at Baseline	Concern Number
Patient Sex	Concern Name
Male	Concern Category Value
Female	Physical and functional well-being
Cancer Stage	Social care and well-being
Stage 0	Psychological/emotional/spiritual well-being
Stage I	Treatment related
Stage II	Other
Stage III	
Stage IV	Concern discussed
Consultant Number	Yes
Number of PCI Items Selected	No
Duration of Consultation (s)	Duration of Consultation (s)
Number of Concerns Discussed	Timestamp First Mention of Each Concern (s)
% PCI Concerns Selected That Were Discussed	Who Mentioned Concern
Who Initiated Concern	Patient
Patient	Consultant
Consultant	Not Discussed
Timestamp First Concern Mentioned (s)	Discussed From 1 (s)*
Total Time Spent Discussing Concerns (s)	Discussed Until 1 (s)*
% Of Consultation Spent Discussing Concerns	Time Spent Discussing Concern 1 (s)*
	Total Time Spent Discussing Individual Concern (s)

Thirty-six tapes were recoded 10 days later to calculate an intra-rater test–retest of the coding technique applied.

### Data analysis

Data analysis was conducted using STATA software [20]. Univariate t-tests were utilised to investigate differences in means between PCI and non-PCI groups. A Fisher’s Exact test assessed the prevalence of psychological concerns compared to all other concerns. A Pearson’s Chi-Square test assessed the relationship between trial group and who initiated the first concern.

To predict differences in the number of concerns raised between trial groups a mixed effects linear regression model (using STATA *mixed* routine) was utilised, estimating the effect of the PCI after adjusting for fixed-effects and clustering of patients within consultant(i.e., a 2-level model). To predict differences in who initiates the first concern in HNC consultation a mixed effects logistic regression model (using STATA *melogit* routine with 10 integration points) was utilised, estimating the effect of the PCI after adjusting for fixed and clustering effects. This model consisted of 3 levels: concerns, patients, and consultants. Relevant data from the Patient File was considered as fixed-effect adjusters within both regression models. Alpha level was 0.05 two-sided throughout.

## Results

### Sample overview

Mean consultation duration in the PCI group (*n* = 36) was 618.3 ± 245.4s and in the non-PCI group (*n* = 29) was 426.2 ± 222.9s (Table 1). Consultations in the PCI group were on average 192.1s longer. Participants in the PCI group spent a mean time of 298.8 ± 206.7s discussing concerns, whereas those in the non-PCI group spent a mean time of 169 ± 144.9s. Participants in the PCI group spent on average 129.8s longer discussing concerns, though this difference was not significant (*p* = 0.582).

One hundred and eighty-five concerns were raised in the PCI group and 75 concerns were raised in the non-PCI group. The mean number of concerns raised per consultation in the PCI group was 5.1 (s.d = 3.1) and 2.6 (s.d = 1.6) in the non-PCI group. Participants in the PCI group raised on average 2.5 more concerns, t(65) = 3.9, *p* < 0.001. The type of concern raised in PCI and non-PCI groups also differed, with the greatest variation being the number of psychological concerns raised in the PCI group, at 28 (15.1%), compared to only 3 (4%) in the non-PCI group, *p* < 0.05 (Table 2).

**Table 2:** Descriptive statistics and frequencies (*n* = 65)

	PCI (*n* = 36)	Non-PCI (*n* = 29)	Total (*n* = 65)
Descriptive Statistics			
Age at Baseline Clinic			
Mean (SD) [Range]	62.8 (10.4) [35;84]	57.1 (10.5) [28;76]	60.2 (10.7) [28;84]
Duration of Consultation (s)			
Mean (SD) [Range]	618.3 (245.4) [300;1200]	426.2 (222.9) [120;960]	532.6 (252.9) [120;1200]
Number of Concerns Discussed			
Sum Mean (SD) [Range]	185 5.1 (3.1) [1;11]	75 2.6 (1.6) [0;7]	260 3.9 (2.9) [0;11]
Time of First Concern (s)			
Mean (SD) [Range]	38.5 (23.5) [7;105]	38.9 (19.8) [8;90]	38.7 (21.8) [7;105]
Total Time Discussing Concerns (s)			
Mean (SD) [Range]	298.8 (206.7) [11;874]	169.0 (144.9) [0;605]	240.9 (191.8) [0;874]
Frequencies			
Initiated First Concern, n (%)			
Patient	31 (86.1%)	21 (72.4%)	
Consultant	5 (13.9%)	7 (24.1%)	
No Concerns Raised	0 (0.0%)	1 (3.4%)	
Gender, n (%)			
Female	13 (68.4%)	6 (31.6%)	19 (100%)
Male	23 (50.0%)	23 (50%)	46 (100%)
Overall stage, n (%)			
Stage 0	1 (100%)	0 (0.0%)	1 (100%)
Stage I	10 (41.7%)	14 (58.3%)	23 (100%)
Stage II	4 (66.7%)	2 (33.3%)	6 (100%)
Stage III	8 (66.7%)	4 (33.3%)	12 (100%)
Stage IV	13 (59.1%)	9 (40.9%)	22 (100%)
Category of Concern Discussed, n (%)			
Physical & Functional Well-being	124 (67%)	48 (64%)	
Social Care and Well-being	10 (5.4%)	8 (10.6%)	
Psychological/Emotional/ Spiritual Well-being	28 (15.1%)	3 (4%)	
Treatment Related	5 (2.7%)	4 (5.4%)	
Other	18 (9.7%)	12 (16%)	
Total	185 (100%)	75 (100%)	

Patients in the PCI group initiated the first concern 31 (86.1%) times, with the consultant initiating the first concern 5 (13.9%) times. In the non-PCI group, patients initiated the first concern 21 (72.4%) times, with the consultant initiating the first concern 7 (24.1%) times. On one occasion there were zero concerns raised in the non-PCI group. There was no significant relationship between trial group and who initiated the first concern (chi-square = 2.53, df2, *p* = 0.28).

#### Objective 1

Predictors of Number of Concerns Raised (1 = PCI, 0 = non-PCI).

The mixed effects multiple regression model (Table 3) was found to significantly predict the number of concerns raised during a consultation, χ^2^(6) = 65.53, *p* < 0.001. The regression coefficient for PCI group (β = 1.3, 95% CI [0.192, 2.395]) indicated that participants in the PCI group were predicted to raise, on average, an increase of 1.3 concerns per consultation when compared with non-PCI patients. There was also an impact of who initiated the first concern on the total number of concerns raised within the consultation (β = -1.06, CI [-2.06, -0.067]) indicating that when the patient initiated the first concern there is predicted to be, on average, 1.06 more concerns discussed. Duration of consultation (*log*) had an effect in the expected direction (β = 2.91, CI [1.96, 3.85]), where longer consultations had more concerns. There was no effect of patient age (*p* = 0.083), patient stage of illness (*p* = 0.054), or patient sex (*p* = 0.709) on the number of concerns raised.

**Table 3:** Mixed effects multiple regression predicting number of concerns raised (*n* = 65)

**Concerns Raised**	**Coeff**	**Robust Std. Err**	**P >|z|**	**[95% Conf. Interval]**		
Consultation Duration	2.907	.4828	.001	1.961	3.854	
Patient Age	-.0222	.0128	.083	-.0474	.0029	
Cancer Stage	.2732	.1417	.054	-.0046	.5509	
Who initiated Concern ^A^	-1.064	.5084	.036	-2.060	-.0674	
Doctor						
Patient Sex ^B^	.2983	.8006	.709	-1.271	1.867	
Male						
Trial Group ^C^	1.295	.5615	.021	.1942	2.395	
PCI						
Constant	-14.03	3.233	.001	-20.37	-7.696	
**Random-effects Parameters**	**Estimate**	**Robust Std. Err**		**[95% Conf. Interval]**		
Consultant						
Var (Constant)	.3289	.3122		.0512		2.114
Var (Residual)	3.607	.8816		2.234		5.823

Reference Category ^A^ = Patient

Reference Category ^B^ = Female

Reference Category ^C^ = non-PCI trial group

Random effect estimates were analysed to investigate the contribution of clustering to explain the variance of the dependent variable. Analysis indicated there to be no significant effect for clustering for consultant (t = 1.05). Scatterplot graphs indicating the number of concerns raised against log consultation duration (one of the strongest independent variables associated with number of concerns) for each consultant, provided some further evidence that the clustering effect was not great as virtually all (except for two) of the slopes in individual scatterplots were positive (Appendix 3).

#### Objective 2

Predictors of Who Initiated the First Concern (1 = Consultant, 0 = Patient).

The mixed effects logistic regression model (Table 4) was found to significantly predict who initiated the first concern in the consultation, χ^2^(11) = 21.97, *p* < 0.05. The regression coefficient for consultation duration (β = 0.001, CI [5.32, 0.003]) (natural score used for ease of explanation) indicated that for every 100 s increase in consultation length it would be predicted that on average the number of concerns would increase by 0.1. Furthermore, it was found that the longer it took to initiate the first concern, the more likely it was the consultant who would raise the first concern (β = 0.012, CI [0.002, 0.023]). Also, the regression coefficients for total time (β = -0.012, CI [-0.020, -0.004]) suggested that as the overall consultation length increased, patients were more likely to raise the first concern. Patient cancer stage was shown to be a significant factor, specifically patients with more severe illness (stage III-IV) were more likely to raise the first concern in a consultation (β = -0.734, CI [-1.43, -0.034]). With regards to the type of concerns discussed, Table 4 indicates that reference to a social care issue as a concern is more likely to be initiated by the consultant (β = 1.58, CI [0.308, 2.85]), whereas concerns out with the PCI checklist (labelled ‘Other’) were more likely to be patient initiated (β = -1.26, CI [-2.23, -0.286]). The sex of the patient (*p* = 0.849) and trial group (*p* = 0.540) were not shown to be significant predictors. Random effect estimates showed no clustering effect for consultants within this model (t = 0.988).

**Table 4:** Mixed effects logistic regression predicting who initiated the first concern (*n* = 65)

**Initiated Concern**	**Coeff**	**Std. Err**	**P >|z|**	**[95% Conf. Interval]**	
Patient Sex ^A^	.0667	.3507	.849	-.6206	.7541
Male					
Cancer Stage ^B^	-.7339	.3572	.040	-1.434	-.0339
Stage III-IV (Severe)					
Patient Age	-.0119	.0146	.415	-.0405	.0167
Consultation Duration	2.854	.0007	.049	5.32e-06	.0028
Category 2 (Social) ^C^	1.581	.6495	.015	.3083	2.854
Category 3 (Psychological)	.0988	.4529	.827	-.7888	.9866
Category 4 (Treatment)	-.3885	.7399	.600	-1.839	1.061
Category 5 (Other)	-1.259	.4964	.011	-2.232	-.2865
Time to first Concern Raised	.0125	.0055	.023	.0017	.0231
Total Time	-.0119	.0043	.005	-.0203	-.0036
Trial Group ^D^	.2576	.4199	.540	-.5655	1.081
PCI					
Constant	.1879	.9571	.844	-1.688	2.064
**Random-effects Parameters**	**Estimate**	**Std. Err**		**[95% Conf. Interval]**	
Consultant Var (Constant)	.1929	.1956		.0265	1.407
Consultant > Patient Var (Constant)	2.04e-33	2.55e-17			

Reference Category ^A^ = Female

Reference Category ^B^ = Stage 0, I, II (Moderate)

Reference Category ^C^ = Category 1 (Physical)

Reference Category ^D^ = non-PCI trial group

## Discussion

This is the first study to investigate the impact of introducing the PCI pre-consultation on specific communication events regarding patient concerns during HNC follow-up consultations. Our previous work has reported the main effects of PCI usage on QoL [24] and an economic evaluation [25]. Results of this current study show a significant difference in number of concerns raised between trial groups, where patients in the PCI group raised on average 2.5 more concerns than non-PCI patients. Results further indicated that, contrary to what was predicted, consultants in the PCI group, on average, did not refer to the specific concerns patients highlighted on the PCI or initiate the first discussion of concern. Rather, patients were more likely to initiate the first concern in both groups. However, there were various predictive factors for whom initiated the first concern, such as: consultation duration, how long it took for the first concern to be raised, category of concern raised, and patient stage of illness.

The current results concur with past research, which found that when patients are free to express themselves, they are likely to initiate between 2–3 concerns [26, 27]. This is consistent with the current study where non-PCI patients raised on average 2.9 concerns. Though, we have extended this research by showing that when introduced to the PCI pre-consultation, patients are enabled to raise more concerns, averaging just over five. Furthermore, regression analysis indicated an increase of 1.3 concerns discussed by patients using the PCI compared with those who did not use the PCI. A possible explanation for this effect may be due to the PCI removing the barriers previously discussed by Brandes et al. [11], and refers specifically to the following: patients not being openly invited to raise concerns, healthcare providers being limited by time, and patients deciding not to raise multiple concerns for fear of disturbing a positive relationship with their consultant. Encouraging patients to express their concerns is important because those who voice less concerns have been shown to experience worsening symptoms, increased anxiety, increased healthcare visits, and reduced satisfaction [10].

Results also suggest that the PCI may influence the type of concern raised by patients. Patients in the PCI group discussed significantly more psychological related concerns, such as anxiety, depression, and fear of recurrence than patients in the non-PCI group. The PCI may have acted as facilitatory tool for patients to highlight concerns that are typically more difficult to broach without having to initiate the discussion. This finding has clinical significance, where Fallowfield et al. [28] reported that detection rates of emotional distress in patients with physical diseases, such as HNC, are low. Detmar et al. [29] states that patients are more likely to discuss concerns related to emotional functioning when health-professionals initiate the discussion. When able to highlight psychological concerns using the PCI, it may allow consultants to recognise emotional issues and enhance their discussion beyond the trivial.

Multiple regression results showed that where patients initiated the first concern, there was an increase in the overall number of concerns raised. This finding suggests that through successfully raising the first concern, patient anxiety in raising additional concerns may decrease. Increased patient confidence has previously been shown to positively impact participation in consultations, patient satisfaction, trust between patient and health-care provider, and reduced levels of anxiety and distress [15, 30]. As PCI patients raised on average more concerns, it suggests they faced less anxiety in raising concerns than patients in the non-PCI group. This finding is supported by Kinnersley et al. [31], who discussed that introducing ‘information needs’ interventions immediately prior to consultations led to reduced patient anxiety. Another important finding was that cancer stage did not predict variance in number of concerns raised. We believe this finding to be positive, as it suggests that consultants exhibit no bias in discussing concerns with patients at different stages of disease.

The multiple-variable logistic regression results revealed that patients were more likely to raise the first concern compared to consultants irrespective of which trial group they were in. This finding was not in the expected direction, where it was predicted that consultants in the PCI group would refer to the concerns highlighted by the patient (raised when completing the PCI) and initiate the first concern accordingly. However, literature suggests that consultants will begin with an open question such as “How can I help you today?” [27], where patients will reply with the initiation of the first concern [32]. Our results show this to be consistent regardless of the PCI being used to highlight concerns pre-consultation. Nonetheless, as there was no significant difference in who initiated the first concern (Table 2), yet a significant difference between patient and consultant in how many concerns were raised overall, perhaps the initiation of the first concern is not an area that needs improving. Rather it is the issue of maintaining momentum of discussing concerns throughout the consultation that requires development. This is potentially an important finding, where it has been shown that new and often more severe concerns are raised further into consultations [10, 33]. We suggest that the PCI contributes to ensuring that after the first concern is discussed, consultants are provided with the information to discuss additional concerns.

### Strengths and clinical significance

First, the choice of cluster design ensured that the possibility of contamination and potential dilution of results due to consultants being in preferred trial groups was avoided. On this, patients were distributed to trial groups dependent on their consultant, therefore there was no patient selection bias as the patient referral process was blinded to consultant trial group. Second, results suggested that in the real world, application of the PCI, would aid HNC patients, by raising an increased number of concerns. Third, the PCI was shown to support patients in raising concerns that may be more difficult to initiate, such as psychological concerns. This has clinical significance, where simply discussing psychological concerns with physicians has been shown to improve overall patient QoL [34]. Furthermore, we have highlighted that the initiation of the first concern may not necessarily require additional close attention by trainers, proposing that ensuring concerns are discussed throughout the consultation is a vital area for increased development. We suggest the PCI is applied as an adjunct to the consulation. Finally, we support past research in showing that the PCI can be implemented without significantly prolonging consultation time [8, 19].

### Limitations and future suggestions

Increased sample size may have allowed for further analysis of the interaction that exists between our predictive variables and the number of concerns discussed. Associated, on one occasion a consultant only met with one patient, which limits the studies ability to conduct a robust cluster analysis. Future studies should aim to recruit more patient participants that are somewhat more evenly distributed across consultants. A key proposal raised by our research is to increase the training that consultants receive before applying the PCI. Although it was used effectively with just 20 min of training [8], we believe there could be improvements with how some consultants chose to utilise the tool. Occasionally consultants did not refer to concerns highlighted on the PCI, which is evidenced through sixty-nine concerns being removed from analysis as they were highlighted pre-consultation, yet not discussed. We suggest that with slightly more training the number of consultants that apply a systematic use of the PCI would increase and benefit the consultation process. Finally, we accept that additional factors (e.g. self-efficacy) may be responsible for biaising our results, that we were unable to control for, or limited statistical power prevented running more complex models.

## Conclusion

The introduction of the PCI pre-HNC consultation has shown to have positive effects on the communication process that takes place between patient and consultant. Most importantly, the PCI has been shown to significantly increase the number of concerns raised in HNC consultations without significantly prolonging consultation time. The PCI has also been shown to support patients in highlighting psychological concerns and aid the continuation of discussing concerns throughout the consultation. We believe that if routinely introduced to HNC centres the PCI would prove to be a beneficial tool that significantly improved the communication process and facilitated discussions about patient concerns between HNC patients and consultant. Readers interested to apply the PCI in their clinical practise are advised to consult the original PCI literature [35].

### Supplementary Information

Below is the link to the electronic supplementary material.

Supplementary file1 (DOCX 296 KB)

## Abbreviations

HNC: Head and Neck Cancer
QPL: Question Prompt List(s)
PCI: Patient Concerns Inventory
QoL: Quality of Life
UW-QOLv4: University of Washington Quality of Life Questionnaire
EQ-5d-5L: European Quality of Life Five Dimension (5 levels)
s: Seconds
SD: Standard Deviation
