# Supplementary material for: Concerns raised by people treated for head and neck cancer: a secondary analysis of audiotaped consultations in a health services follow-up clinic
Source: Support Care Cancer. 2023 Oct 4;31(10):608. doi: 10.1007/s00520-023-08059-w (PMC10550852; doi:10.1007/s00520-023-08059-w)
Supplement: Supplementary file 1 — Supplementary file1 (DOCX 296 KB) [file 520_2023_8059_MOESM1_ESM.docx]

**APPENDICES**

| **Appendix 1.1.** Patient File Coding Scheme* | |  |  | | |  |
| --- | --- | --- | --- | --- | --- | --- |
| **Category** | **Description** | | **How it was coded** | | | **Unit** |
| **Audio Tape Label Name** | Each tape was provided with a label name by Chief Investigator. | | As this was pre-assigned to each audio file it was not coded. Was used as an identifying factor to distinguish tapes. | | | N. A |
| **Trial Group** | Patients were either in the PCI or non-PCI trial group. | | PCI patients coded as 0, non-PCI coded as 1. | | | N. A |
| **Age at Baseline** | Patient age at baseline was provided by the Chief Investigator. | | As this was numerical data pre-assigned to each audio file it was not coded. | | | Years |
| **Patient Sex** | Patient sex was provided by the Chief Investigator. | | Females coded as 0, males coded as 1. | | | N. A |
| **Stage of Illness** | Stage of illness was provided by the Chief Investigator. | | Coded 0-4 for each patient depending on their stage of illness. Code reflected their stage, i.e., 1 = Stage 1. | | | N. A |
| **Consultant Value** | Consultant numbers were provided by the Chief Investigator. | | Consultants were assigned a code 1-15. Code reflected their individual number, i.e., 2 = Consultant 2. | | | N. A |
| **Patient Number** | Patients were provided with a number to assist identification of tapes. | | Coded systematically, i.e., 1 = Patient 1, 2 = Patient 2 etc. | | | N. A |
| **Duration of Consultation** | Length of consultation from start to finish. | | This was rounded to the nearest minute then converted to seconds. | | | s |
| **Total PCI Items Selected** | Number of items that patients had selected pre-consultation using the PCI. | | The number of concerns selected pre-consultation was shown for PCI patients. Non-PCI patients coded as 0 concerns selected as they did not use PCI. | | | N. A |
| **Number of Concerns Discussed** | Number of concerns discussed in each consultation between patient and consultant. | | Concerns coded as ‘discussed’ (see Appendix 1.2) were added up for each patient. | | | N. A |
| **% of Selected PCI Concerns Discussed** | Percentage of how many concerns were discussed compared to the amount selected to be discussed pre-consultation. | | Number of concerns discussed / total PCI items selected = percentage of selected PCI concerns discussed. | | | % |
| **Initiated First Concern** | Who initiated the first concern of the consultation. | | Patient coded as 1, Consultant coded as 2, Not Discussed coded as 3. | | | N. A |
| **Timestamp First Concern** | A timestamp was taken for how long into the consultation it took for the first concern to be mentioned. | | Every concern discussed was provided a timestamp of when it was first mentioned (See Appendix 1.2). From this the timestamp of the first concern was noted again within the Patient File. | | | s |
| **Sum Time Discussing Concerns** | The total time spent discussing concerns in each consultation. | | After each concern was discussed, it was provided with a total time (See Appendix 1.2). Once all concerns had been discussed in a consultation, the total times for each concern were added to provide a sum time discussing concerns in each consultation. | | | s |
| **% of Consultation Discussing Concerns** | Percentage of how much time was spent discussing concerns throughout the consultation. | | Sum time spent discussing concerns / duration of consultation = percentage of consultation discussing concerns. | | | % |
| ***** Each category was coded in a separate column & each patient had a separate row of data. | | | |  |  |  |

| **Appendix 1.2.** Concerns File Coding Scheme ***** | |  |  | | |  |
| --- | --- | --- | --- | --- | --- | --- |
| **Category** | **Description** | | **How it was coded** | | | **Unit** |
| **Audio Tape Label Name** | Each tape was provided with a label name by Chief Investigator. | | As this was pre-assigned to each audio file it was not coded. Was used as an identifying factor to distinguish tapes. | | | N. A |
| **Trial Group** | Patients were either in the PCI or non-PCI trial group. | | PCI patients coded as 0, non-PCI coded as 1. | | | N. A |
| **Patient Number** | Patients were provided with a number to assist identification of tapes. | | Coded systematically, i.e., 1 = patient 1, 2 = patient 2 etc. | | | N. A |
| **Concern Number (PCI) **** | In the PCI group every concern selected pre-consultation was provided a new row. | | Each concern was provided a number, starting at 1 again for the first concern of each patient. If a concern was mentioned out with the pre-selected PCI items, it a provided a new row of data and numbered accordingly. | | | N. A |
| **Concern Number (non-PCI) **** | In the non-PCI group, every time a concern was mentioned in consultation it was provided a new row. | | Each concern that was mentioned was provided a number, starting at 1 again for the first concern of each patient. | | | N. A |
| **Concern Value** | Concern value represented which of the 57 PCI items had been selected/was raised in consultation. | | Concerns were provided a value 1-57, which corresponded to the items on the PCI, i.e., 1 = activity, 2 = appetite. Concerns raised that did not correspond specific concerns on the PCI were coded as Other (57). | | | N. A |
| **Category Value** | Category value represented the 5 types of concerns that could be raised. | | Concerns coded 1-5. 1 = Physical, 2 = Social care, 3 = Psychological, 4 = Treatment, 5 = Other. | | | N. A |
| **Concern Mentioned** | If a concern was mentioned, in that it was stated by either the patient or consultant, it was coded. | | Not mentioned coded as 0, mentioned coded as 1. | | | N. A |
| **Who Mentioned** | Who mentioned the concern was also noted, whether it was the patient, consultant, or not discussed. | | Patient coded as 1, consultant coded as 2, not discussed coded as 3. | | | N. A |
| **Duration of Consultation** | Length of consultation from start to finish. | | This was rounded to the nearest minute then converted to seconds. | | | s |
| **Timestamp First Mention of Each Concern** | Timestamp of when each concern was first mentioned in the consultation. If 5 concerns were mentioned there would be 5 timestamps indicating when each of them was first mentioned. | | Every concern discussed was provided a timestamp of when it was first mentioned. | | | s |
| **Discussed from 1** | If a concern was discussed, in that it was mentioned by someone and picked up by the other, it was coded. | | The time that a concern began to be discussed was coded. | | | s |
| **Discussed Until 1** | When a concern had stopped being discussed, in that there was a clear indicator that they had moved on, it was coded. | | The time that a concern finished being discussed was coded. | | | s |
| **Time Spent 1** | The length of the discussion regarding each concern was coded. Did not have to be a long discussion, could simply be P “I have a dry mouth”, C “Okay, that’s a common side effect of your treatment.”. | | Discussed Until 1 – Discussed From 1 = Time Spent 1. | | | s |
| **Discussed From/Until/Time Spent 2-4. ***** | It was common for concerns to be brought back up later in the consultation. The maximum number of times a concern was discussed on separate occasions in one consultation was 4 times. | | If a concern was discussed for a second, third, or fourth time the same coding scheme was used as the first time it was discussed. *** | | | S |
| **Total Time Discussed** | A total time for how long each concern was discussed for was coded. Note this was the total time for each concern, not the total time spent discussing all concerns in the consultation. | | Time spent 1 + Time spent 2 + Time spent 3 + Time spent 4 = Total time. | | | s |
| ***** Each category was coded in a separate column & each concern had a singular row of data. ****** Coded within the same column. ******* Discussed from 2-4, discussed until 2-4 & time spent 2-4 each has a separate column (total 9). | | | |  |  |  |

| **Appendix 2.** Ethical Approval. |
| --- |


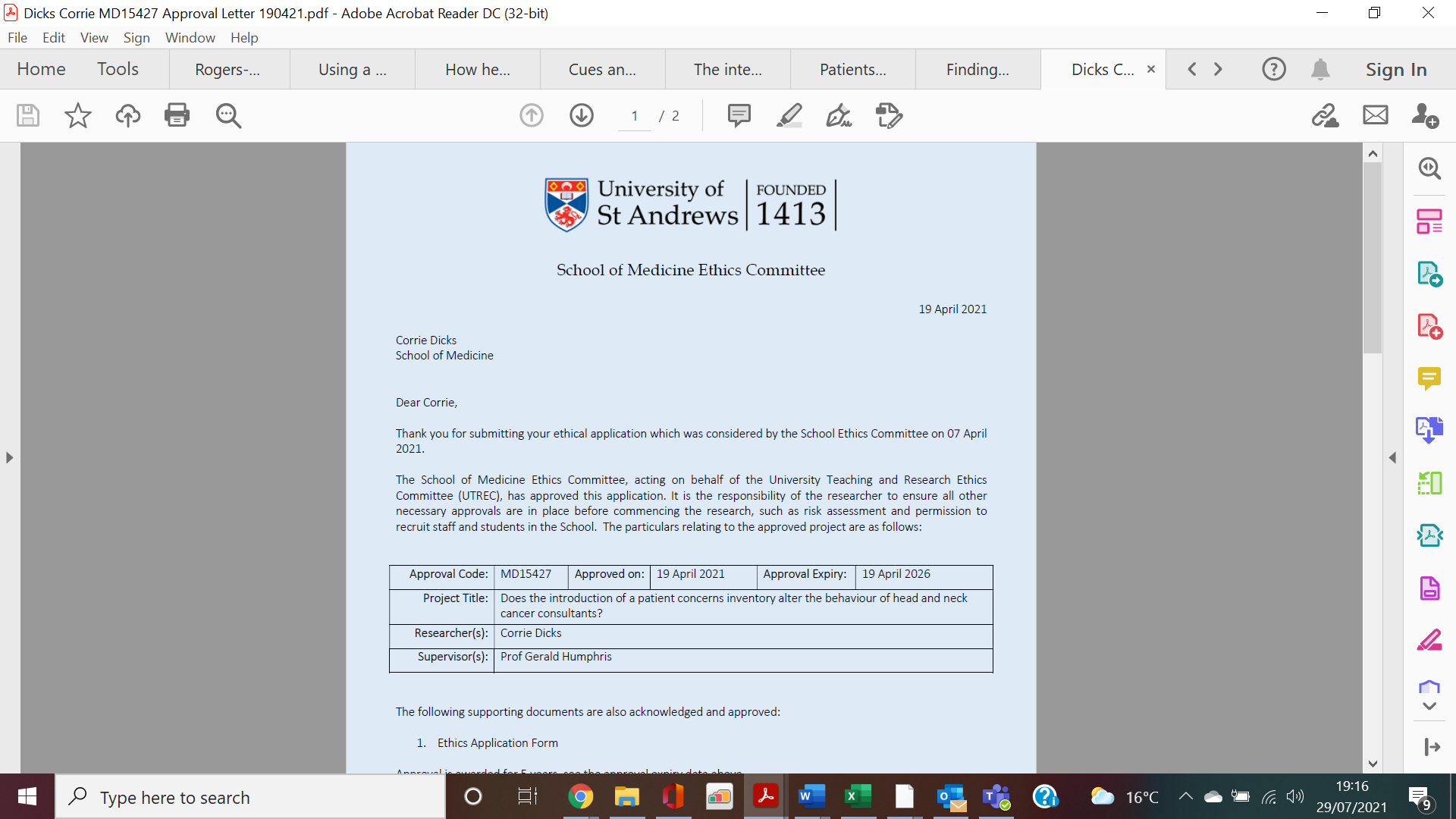

| **Appendix 3.** Number of Concerns Raised Per Consultant plotted against Log (transformed) Duration. |
| --- |

| * Consultant 12 removed due to consulting one patient only. |
| --- |

| **Appendix 4.1.** Multiple Regression Correlations (N=65). | | | | | | | | | | | | | |
| --- | --- | --- | --- | --- | --- | --- | --- | --- | --- | --- | --- | --- | --- |
| *Pearson Correlation* | | **Consultation Duration** | | | **Patient Age** | | **Treatment Stage** | | **Who Initiated Concern** | | **Patient Sex** | | **Trial Group** |
| **Consultation Duration** |  | | 1 | .169 | | .013 | | -.199 | | .034 | | -.368^**^ | |
| **Patient Age** |  | | .169 | 1 | | -.171 | | -.114 | | -.059 | | -.293^*^ | |
| **Treatment Stage** |  | | .013 | -.171 | | 1 | | .035 | | .203 | | -.083 | |
| **Who Initiated Concern** |  | | -.199 | -.114 | | .035 | | 1 | | .145 | | .166 | |
| **Patient Sex** |  | | .034 | -.059 | | .203 | | .145 | | 1 | | .159 | |
| **Trial Group** |  | | -.368^**^ | -.293^*^ | | -.083 | | .166 | | .159 | | 1 | |
| *. Correlation is significant at the 0.05 level (2-tailed).  **. Correlation is significant at the 0.01 level (2-tailed). | | | | | | | | | | | | | |
|  | | | | | | | | | | | | | |

| **Appendix 4.2.** Logistic Regression Correlation Matrix (N=65). | | | | | | | | | | | | | | |
| --- | --- | --- | --- | --- | --- | --- | --- | --- | --- | --- | --- | --- | --- | --- |
| *Pearson Correlation* | | **Patient Sex** | | **Treatment Stage** | | **Patient Age** | | **Duration of Consultation** | | **Time to First Concern Raised** | | **Total Time** | | **Trial Group** |
| **Patient Sex** |  | | 1 | .203 | -.059 | | .034 | | .093 | | .040 | | .159 | |
| **Treatment Stage** |  | | .203 | 1 | -.171 | | .013 | | .057 | | .013 | | -.083 | |
| **Patient Age** |  | | -.059 | -.171 | 1 | | .169 | | .008 | | .107 | | -.293^*^ | |
| **Duration of Consultation** |  | | .034 | .013 | .169 | | 1 | | -.150 | | .864^**^ | | -.368^**^ | |
| **Time to First Concern Raised** |  | | .093 | .057 | .008 | | -.150 | | 1 | | -.196 | | .144 | |
| **Total Time** |  | | .040 | .013 | .107 | | .864^**^ | | -.196 | | 1 | | -.326^**^ | |
| **Trial Group** |  | | .159 | -.083 | -.293^*^ | | -.368^**^ | | .144 | | -.326^**^ | | 1 | |
| *. Correlation is significant at the 0.05 level (2-tailed). | | | | | | | | | | | | | | |
| **. Correlation is significant at the 0.01 level (2-tailed). | | | | | | | | | | | | | | |
